# Exposure to Antineoplastic Agents Induces Cytotoxicity in Nurse Lymphocytes: Role of Mitochondrial Damage and Oxidative Stress

**Published:** 2018

**Authors:** Mohmmad Ali Eghbal, Elham Yusefi, Maria Tavakoli-Ardakani, Maral Ramazani, Mohammad Hadi Zarei, Ahmad Salimi, Jalal Pourahmad

**Affiliations:** a *Department of Pharmacology and Toxicology, Faculty of Pharmacy, Tabriz University of Medical Sciences, Tehran, Iran. *; b *Department of Pharmacology and Toxicology, Faculty of Pharmacy, Shahid Beheshti University of Medical Sciences, Tehran, Iran. *; c *Department of Pharmacology and Toxicology, School of Pharmacy, Ardabil University of Medical Science, Ardabil, Iran.*

**Keywords:** Nurses, Human Lymphocytes, Inhalation Exposure, Mitochondrial Damage, Antineoplastic Drugs

## Abstract

Cytotoxicity and mitochondrial parameters were studied in isolated lymphocytes and their mitochondria obtained from occupationally exposed nurses through inhalation exposure to antineoplastic drugs and results were compared to those of unexposed nurses. The group of occupationally exposed nurses consisted of 50 individuals ranging in age from 30 to 35 years. The control group included 50 nurses who were not occupationally exposed to the preparation and handling of antineoplastic drugs and their anthropometric and biochemical characteristics were similar to those of the expose group. All cytotoxicity and mitochondrial parameters evaluated in exposed group were significantly increased (*P* < 0.05) compared to the unexposed control group. Finally, the results of our study suggest that using antioxidant, mitochondrial and lysosomal protective agents can be promising drug candidates for the hospital staff in the risk of exposure to exposure to antineoplastic drugs.

## Introduction

Antineoplastic drugs are widely used agents for the treatment of cancer. most of these agents act by interfer­ing directly with the deoxyribonucleic acid of tumour cells and thereby intercept their growth (1). Unfortunately, antineoplastic drugs are generally non-selective and therefore healthy cells may also be damaged which, in turn, results in toxic effects. Given this, there is a risk to healthcare individuals who handle, prepare, and administer antineoplastic drugs (2). Numerous investigations have examined antineoplastic drug contamination in healthcare instruments. Studies have demonstrated surface contamination of countertops, cabinets and floors within the drug preparation area (3). Traceable levels of environmental drug contamination have also been found in patient care areas. In the absence of any current occupational exposure limits for antineoplastic drugs, it is therefore important to minimize contamination (3). More investigations revealed that these surface contaminations were within a healthcare facility the pharmacy, where the drugs are provided, and the administration units where the prepared drugs are given to patients (4). The emphasis on these places is warranted since direct handling of the drugs is anticipated during both preparation and administration (5, 6).

Employees in oncological units may experience occupational exposure to low doses of anticancer drugs (7). The International Agency for Research on Cancer (IARC) has classified many anticancer drugs as class 1 human carcinogens such as cyclophosphamide, etoposide, busulfan, melfalan, class 2A probably carcinogenic to humans such as azacitidine, cisplatin, doxorubicine and class 2B possibly carcinogenic to humans such as bleomycin, dacarbazine, mitoxantrone, mitomycin (8). Occupational exposure to anticancer drugs has been shown to result in increased total chromosomal aberrations (9-11). Interestingly, nurses occupationally exposed to anticancer drugs showed an elevated level of not only chromatid-type aberrations (CTA), but also chromosome-type aberrations (CSA), such as chromosome breaks, which is typical of radiation exposure (12).

Cancer is one of the leading causes of death in the world. A large percentage of patients are diagnosed at an advanced stage, making the removal of tumors in this population problematic. Therefore, early stage detection would be helpful in reducing cancer mortality (13). For this reason, a well-established assessment model would greatly benefit patients, clinicians, and researchers because it would allow individuals at high risk to be identified at the earliest stages. Accurately assessing cancer risk in average- and high-risk individuals is crucial to controlling the suffering and death due to cancer. Cancer prediction proceedings provide an important approach to assessing risk by identifying individuals at high risk (13). 

Human peripheral blood lymphocytes (PBL) are extensively used in biomonitoring of populations exposed to various mutagenic or carcinogenic compounds (14). This is because of the ease of sampling, the possibility of obtaining large numbers of scorable cells (15). The conceptual basis for using of toxicity in PBL as a biomarker has been the hypothesis that the extent of genetic damage in PBL reflects similar events in the precursor cells for carcinogenic processes in the target tissues (16). The aim of this work was to evaluate cellular and mitochondrial toxicity parameters such as cytotoxicity, reactive oxygen species (ROS), mitochondrial membrane potential (MMP), lysosomal integrity, glutathione levels, lipid peroxidation in the blood lymphocytes of oncology nurses occupationally exposed through inhalation exposure to anticancer drugs and compare the findings with those of unexposed hospital nurses. 

## Experimental

*Cellular Assessments*

*Blood samples*

All blood samples (exposed nurses = 50 and unexposed nurses = 50) were acquired and approved by Blood Administration Center. Exposed nurses were exposed with anti-cancer drugs such as 5- Fluoro Uracil, Methotrexate, Cisplatin, Vinblastine and Tamoxifen. The studies were performed at the school of Pharmacy, Shahid Beheshti University of Medical Sciences, under the guidance of an expert physician. This study was approved by Shahid Beheshti University of Medical Sciences, and all healthy individuals signed an informed consent form.

*Lymphocytes isolation*

Lymphocytes were collected from nurses occupationally exposed to anticancer drugs and unexposed nurses at age range 30 to 35 years old whose anthropometric and biochemical characteristics were similar to those of the expose group. Blood was obtained from, non-smoking volunteers, who showed no signs of infection disease symptoms at the time that the blood samples were collected. Lymphocytes were isolated using Ficoll Paque Plus according to the manufacturerꞌs instructions. The obtained lymphocytes were suspended in RPMI1640 medium with L-glutamine and 10% FBS. The final lymphocytes account used in the experiments was 1 × 10^6^ cells/mL. The viability of the lymphocytes was over 95% (17, 18). 

*Determination of ROS*

To determine the rate of lymphocytes ROS generation, dichlorofluorescein diacetate (DCFH-DA, 1.6 µM) was added to the lymphocytes. It penetrates lymphocytes and becomes hydrolyzed to non-fluorescent dichlorofluorescein (DCFH). The latter then reacts with ROS to form the highly fluorescent dichlorofluorescein (DCF), which effluxes the cell. The fluorescence intensity of DCF was measured using a Shimadzu RF5000U fluorescence spectrophotometer. Excitation and emission wavelengths were 500 nm and 520 nm, respectively. The results were expressed as fluorescent intensity per 10^6^ cells (17, 19). 

*MMP Assay*

Mitochondrial uptake of the cationic fluorescent dye, rhodamine 123 (1.5 µM), has been used for the estimation of mitochondrial membrane potential. The amount of rhodamine 123 remaining in the incubation medium was measured fluorimetrically using a Shimadzu RF5000U fluorescence spectrophotometer set at 490 nm excitation and 520 nm emission wavelengths. The capacity of mitochondria to take up the rhodamine 123 was calculated as the difference (between control and treated cells) in rhodamine 123 fluorescence (17, 20).

*Lysosomal Membrane Integrity Assay*

Lymphocytes lysosomal membrane stability was determined from the redistribution of the fluorescent dye, acridine orange. Aliquots of the cell suspension (0.5 mL) that were previously stained with acridine orange (5 µM) were separated from the incubation medium by 1 min centrifugation at 1000 rpm. Cell washing process was carried out twice to remove the fluorescent dye from the media. Acridine orange redistribution in the cell suspension was then measured fluorimetrically using a Shimadzu RF5000U fluorescence spectrophotometer set at 495 nm excitation and 530 nm emission wavelengths (17, 21). 

*Lipid Peroxidation*

Evaluation of lipid peroxidation in lymphocytes was conducted by determining the amount of thiobarbituric acid reactive substances (TBARS) formed during the decomposition of lipid hydro peroxides by following the absorbance at 532 nm in a Beckman DU-7 spectrophotometer (17).

*GSH and GSSG*

GSH and GSSG were determined according to the spectrofluorometric method (22). Each sample was measured in quartz cuvettes using a fluorimeter set at 350 nm excitation and 420 nm emission wavelengths.

*ADP/ATP ratio Assay*

Changes in the ADP/ATP ratio have been used to differentiate modes of cell death and viability. ADP/ATP ratio was assessed by ADP/ATP Ratio Assay kit (MAK135 sigma, USA) in lymphocytes using luminometer. ADP/ATP ratio was performed according to the manufacturer’s instructions (23).

*Mitochondrial Assessments*

*Lymphocytes Lysis and Isolation of Mitochondria*

Mitochondria were isolated from the lymphocytes by mechanical lysis and differential centrifugation. Briefly, lymphocytes were washed with cold PBS at 4 ºC and centrifuged at 450 × g. The pellet was resuspended in cold isolation buffer (75 mmol/L sucrose, 20 mmol/L HEPES, 225 mmol/L mannitol, 0.5 mmol/L EDTA, pH 7.2), and the cells were disrupted by homogenization. Nonlysed lymphocytes and nuclei were spun down by centrifugation at 750 × g for 20 min. The supernatant was further spun at 10,000 × g for 10 min twice. The pellet, designated as the mitochondrial fraction, was suspended in assay buffer (140 mmol/L KCl, 10 mmol/L NaCl, 2 mmol/L MgCl_2_, 0.5 mmol/L KH_2_PO_4_, 20 mmol/L HEPES, 0.5 mmol/L EGTA; adjusted to pH 7.2 with KOH) (24, 25).

*Succinate Dehydrogenases Activity*

Complex II Enzyme Activity was measured by Complex II Enzyme Activity Microplate Assay Kit (ab109908, United Kingdom). Each well in the kit has been coated with an anti-Complex II monoclonal antibody (mAb) which purifies the enzyme from a complex sample. After this in-well purification the production of ubiquinol by the enzyme is coupled to the reduction of the dye DCPIP (2,6-diclorophenolindophenol) and a decreases in its absorbance at 600 nm, which in turn recycles the substrate ubiquinone (26).

*Mitochondrial Swelling Assay*

Mitochondria suspensions (at 100 µg protein per well) were incubated in 96-well plates at 25 ºC in swelling buffer (140 mmol/L KCl, 10 mmol/L NaCl, 2 mmol/L MgCl_2_, 0.5 mmol/L KH_2_PO_4_, 20 mmol/L HEPES, 0.5 mmol/L EGTA; adjusted to pH 7.2 with KOH) supplemented with 1 mg/mL rotenone and 10 mmol/L succinate. Mitochondrial swelling was measured spectrophotometrically in duration 1 h. Mitochondrial swelling results in a decrease in absorbance monitored at 540 nm (27).

*Cytochrome C Release Assay*

The concentration of cytochrome C was determined through using the Quantikine Human Cytochrome C Immunoassay kit (Minneapolis, Minn). Cytochrome C measurement was performed according to the manufacturer’s instructions (28).

*Statistical Analysis*

Statistical analyses were performed by GraphPad Prism 6. Results were presented as mean ± SED. Assays were performed in triplicate and the mean was used for statistical analysis. All graphs were expressed as mean ± SEM and *P* < 0.05 was considered statistically significant. 

## Results

*ROS formation*

**Figure 1:**
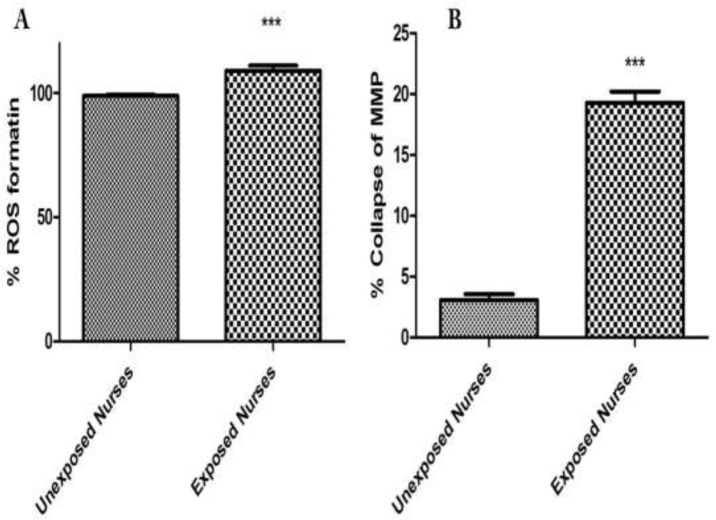
ROS formation (A) and MMP collapse (B) assay in lymphocytes. ROS formation was measured fuorometrically using DCF-DA as described in materials and methods. Also collapse of MMP was measured by rhodamine 123 as described in materials and methods. Each bar represents mean   SEM. Each group consisted of 50 individuals. *** *p* < 0.001 significant difference compared to the unexposed group

There is equilibrium between ROS formation and endogenous antioxidant defense mechanisms, but if this balance is disturbed, it can produce oxidative stress. As shown in Figure 1A, isolated lymphocytes from oncology nurses in comparison with healthy unexposed individuals showed significant increase at ROS formation. 

*MMP Collapse*

To monitor the mitochondrial membrane potential, lymphocyte mitochondria were stained with rhodamine 123. Isolated lymphocytes from oncology nurses in comparison with healthy unexposed individuals showed a significant collapse at the mitochondrial membrane potential. These changes were in the accordance with the order of ROS formation of the isolated lymphocytes from oncology nurses (Figure 1B).

*Lysosomal Integrity*

**Figure 2:**
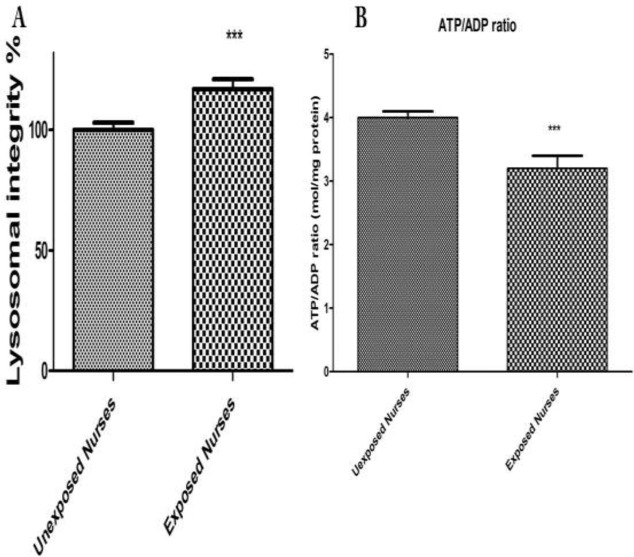
Lysosomal membrane damage (A) and ATP level (B). Human lymphocytes (10^6^ cells/mL) were suspended in the RPMI 1640 at 37 ^o^C. Lysosomal membrane damage was determined as the difference in redistribution of acridine orange from lysosomes into cytosol between both groups. ATP/ADP ratio were determined by Luciferin /Luciferase assay System as described in Materials and Methods. Each bar represents mean   SEM. Each group consisted of 50 individuals. *** *p* < 0.001 significant difference compared to the unexposed group

As shown in Figure 2A there was a loss of lysosomal membrane integrity measured by redistribution of fluorescent dye acrdine orange in the lymphocytes isolated from nurses occupationally exposed to anticancer drugs. While no significant lysosomal damage was detected in lymphocytes isolated from healthy unexposed nurses.

*ADP/ATP ratio*

ATP and ADP were measured using a bioluminescence assay based on luciferin/luciferase reaction. The ADP and ATP were measured at both groups (Figure 2B). As shown in Figure 2B there was a significant decrease in ATP and a significant increase in ADP levels only in lymphocytes isolated from oncology nurses (*P* < 0.05) while no significant changes at ADP/ATP ratio observed in healthy unexposed staff lymphocytes. 

*Lipid Peroxidation and Glutathione Levels*

**Figure 3:**
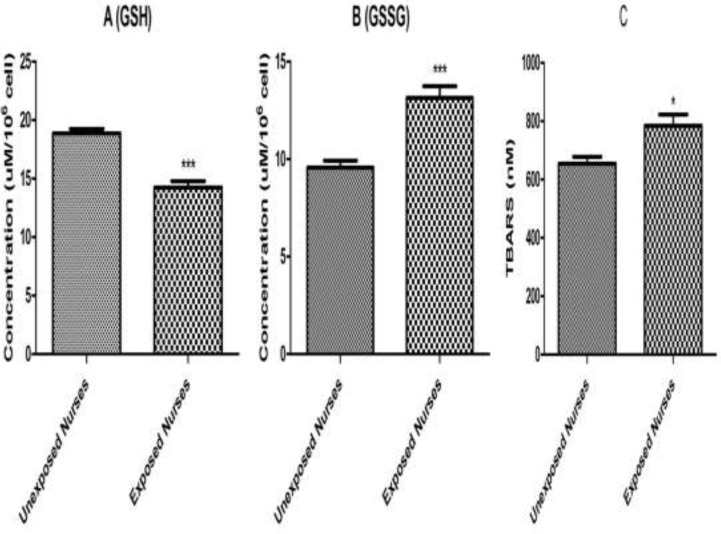
GSH (A), GSSG (B) and MDA (C). Lymphocytes (10^6^ cells/mL) were suspended in the RPMI 1640 at 37 ^o^C. Intracellular GSH and extra cellular GSSG were fluorimetrically determined. Also, TBARS formation was expressed as nM concentrations. Each bar represents mean   SEM. Each group consisted of 50 individuals. *** *p *< 0.001 significant difference compared to the unexposed group

Lipid per-oxidation in lymphocytes was determined by measuring MDA formation. As shown in Figure 3, significant MDA formation only observed in the lymphocytes isolated from nurses occupationally exposed to anticancer drugs. While no significant MDA formation was detected in lymphocytes isolated from healthy unexposed nurses.

In addition, as shown in Figure 3 reduced (GSH) and oxidized (GSSG) glutathione levels were determined under the same experimental conditions and correlated with MDA formation and other measured parameters. A significant decrease in reduced glutathione (GSH) level was observed in the lymphocytes isolated from nurses occupationally exposed to anticancer drugs followed by a significant increase of oxidized (GSSG) glutathione levels compared to those of healthy unexposed staff under the similar condition.

*Mitochondrial Assessment*

*SDH Activity*

**Figure 4:**
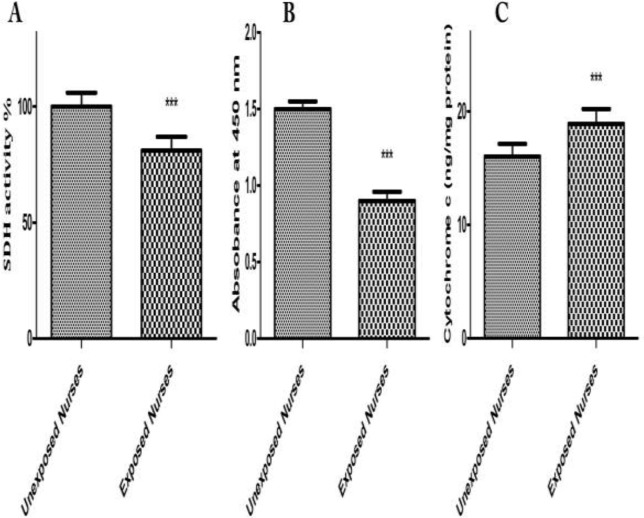
SDH activity (A), mitochondrial swelling (B) and cytochrome c (C). Mitochondrial SDH activity were measured by kit at 1 h. Mitochondrial swelling was measured at 30 min, by determination of absorbance decline at 540 nm. Cytochrome c release was measured by ELISA kit as described in Experimental methods. Each bar represents mean   SEM. Each group consisted of 50 individuals*** *p *< 0.001 significant difference compared to the unexposed group

Evaluations of SDH Activity in isolated mitochondria obtained from both oncology and healthy nurses were carried out by studying succinate dehydrogenase activity using the Complex II Enzyme Activity Microplate Assay Kit. Succinate dehydrogenase activity significantly reduced only in mitochondria from oncology but not in healthy unexposed nurses’ lymphocytes (Figure 4A). 

*Mitochondrial Swelling*

Mitochondrial swelling has been considered as an indication of the opening of the MPTP, which results in depolarization of mitochondrial membrane potential. We observed that mitochondrial swelling occurred only in mitochondria obtained from oncology but not healthy unexposed nurses’ lymphocytes. We also recorded mitochondrial membrane potential decrease using rhodamine 123 and ROS formation using DCF-DA in the isolated mitochondria obtained from blood lymphocytes of both groups (data not shown) (Figure 4B). 

*Cytochrome C Release*

The process of cell death involves the release of cytochrome C from the mitochondria, which subsequently causes apoptosis. Our results showed significant mitochondrial swelling and collapse of the mitochondrial membrane potential only in mitochondria isolated from oncology nurses’ lymphocytes (data not shown). These events could result in mitochondrial permeability transition and release of cytochrome C from mitochondria into the cytosolic fraction. As shown in Figure 4C, significant release of cytochrome C was shown in mitochondria isolated from nurses’ lymphocytes compared to unexposed blood lymphocyte mitochondria.

## Discussion

We hypothesized that exposure to antineoplastic drugs leads to increased toxicity in blood lymphocytes in the hospital nurses or staff occupationally exposed to anticancer drugs. Through observation and biochemical experiments, our study results proved that toxicity parameters increased in blood lymphocytes of exposed group compared to those of unexposed nurses. Through different studies show that surface contamination, and accordingly the potential for occupational exposure to antineoplastic drugs, occurs at every stage of the hospital medication system (2). Those job categories most likely in risk of exposure are nurse, pharmacy technician, pharmacy receiver, and pharmacist. Over 11 job categories (not including housekeeping) per site are potentially at risk of exposure. Several studies have shown that exposure to antineoplastic drugs can cause toxic effects (2). Dermal exposure has been suggested to be the main route of exposure (29). In a recently study, was assessed dermal exposure to cyclophosphamide. Surprisingly, exposure levels detected on the hands during nursing tasks were higher than those measured in the hospital pharmacy during preparation of cyclophosphamide. The positive urine samples with detectable cyclophosphamide and other anti-neoplastic drugs were observed in hospital personals (29). Also studies show that the prevalence of needle stick injury (NSI) in Iranian nurses is high that likelihood exposure to anti-neoplastic drugs has increased (30). 

ROS formation was compared between occupationally exposed nurseꞌs lymphocytes and unexposed. Referring to the results of ROS formation status in the present study, it was shown that cellular and mitochondrial ROS formation was significantly increased in the lymphocytes of occupationally exposed group, compared to those of unexposed control (*P* < 0.05) (Figure 1A). Neoplastic disease studies revealed that treatment with anti-neoplastic drugs increases ROS and reduces plasma levels of vitamins C and E as well as of glutathione peroxidase (31). Consumption of variety of anti-cancer drugs have been associated with oxidative stress. For example, cisplatin induces formation of reactive oxygen species (ROS) in renal proximal tubular cell mitochondria, eliciting oxidative alterations in lipids, proteins, and DNA of this organelle (32), while doxorubicin induced cytotoxicity has been associated with ROS production and in particular to presence of the superoxide anion radical and of hydrogen peroxide in cardiomyocytes (33). This drug is also able to produce reactive nitrogen species. The oxidant RNS such as peroxynitrite is known to induce protein oxidation and nitration in the absence of GSH, eliciting mitochondrial dysfunction and eventually leading to irreversible damage and severe loss of cellular ATP (34). A recent study has also indicated that exposed nurses to antineoplastic drug suffer from oxidative stress compared to unexposed nurses (35). It is worth noting that both drugs are prepared, handled, and administered by nursing personnel in hospitals. Our results proved that content of glutathione and ATP has decreased in occupationally exposed nurses lymphocytes in comparison to unexposed nurses (Figure 3 and Figure 2B). The decreases in lipid peroxidation level found in our study may be explained by an increase in ROS formation produced by anti-neoplastic drugs in occupationally exposed nurseꞌs lymphocytes, such as superoxide anion and hydrogen peroxide, which are known to attach to membrane lipids, initiating lipid peroxidation. Similarly, increased ROS formation may directly oxidize the prosthetic protein group or else reacts directly with the peptide chain, leading to conformational and functional changes in cellular organelles such as mitochondria and lysosomes (36-38). The results of this study also showed occurrence of mitochondrial and lysosomal damages in occupationally exposed nurseꞌs lymphocytes, compared to the control group (*P* < 0.05) (Figure 1B and Figure 2A).

Previous studies indicated an increased risk of a prolonged time to pregnancy among nurses with relatively high exposure to antineoplastic agents compared with referent nurses (39). In addition to the toxicity to reproduction system, antineoplastic drugs have been found to have carcinogenic potential. Nine antineoplastic drugs have been classified by IARC to be carcinogenic to humans. The leukemia risk of an oncology nurse after 40 years of dermal exposure to cyclophosphamide was estimated to be on average of 0.27 (range = 0 - 40) extra cases per million oncology nurses (40).

Mitochondria perform significant roles in cellular energy metabolism, free radicals generation, control of cell death, growth, development, integration of signals from mitochondria to nucleus and nucleus to mitochondria, and various metabolic pathways (41, 42). Introduction of mtDNA mutations in transformed cells has been associated with increased ROS production and tumor growth. Studies revealed that increased and altered mtDNA plays a role in the development of cancer (43). Mitochondrial alterations have been identified in several cancers (44). An increase in mtDNA copy number was observed in patients with a higher risk for breast cancer (45). Our results on isolated mitochondria obtained from occupationally exposed nurseꞌs lymphocytes showed significant mitochondrial toxic changes such as mitochondrial swelling, inhibition of SDH and cytochrome C release (Figure 4). Mitochondrial alterations have been associated with some forms of cancer and an increased risk of certain age-related disorders such as heart disease, Alzheimer disease, Parkinson disease and immunotoxicity (46). Additionally, our results suggest that the gradual and progressive occurrence of these alterations in exposed oncology drugs may play a role in progression of mentioned diseases. 

Our results showed that occupationally exposed nurseꞌs lymphocytes were more susceptible to oxidative stress than unexposed nurses. Finally, isolated mitochondria from lymphocytes could be a biomarker for determination people at risk of oxidative diseases such as cancer and aging.
